# Identifying Needs: a Qualitative Study of women’s Experiences Regarding Rapid Genetic Testing for Hereditary Breast and Ovarian Cancer in the DNA BONus Study

**DOI:** 10.1007/s10897-016-9996-z

**Published:** 2016-07-28

**Authors:** Mirjam Tonheim Augestad, Hildegunn Høberg-Vetti, Cathrine Bjorvatn, Ragnhild Johanne Tveit Sekse

**Affiliations:** 1 Western Norway Familial Cancer Center, Haukeland University Hospital, Haukelandsveien 22, P.O. Box 1400, N-5021 Bergen, Norway; 2 Center for Medical Genetics and Molecular Medicine, Haukeland University Hospital, Bergen, Norway; 3 Department of Clinical Science, University of Bergen, Bergen, Norway; 4 Department of Obstetrics and Gynecology, Haukeland University Hospital, Bergen, Norway

**Keywords:** Rapid genetic testing, BRCA 1/2, Genetic information, Newly-diagnosed breast and ovarian cancer, Personalized support and counselling

## Abstract

Genetic testing for hereditary breast and ovarian cancer is increasingly being offered in newly diagnosed breast and ovarian cancer patients. This genetic information may influence treatment decisions. However, there are some concerns that genetic testing offered in an already vulnerable situation might be an extra burden to these women. The aim of this study was to explore the experiences of women who had been offered and accepted genetic testing when newly diagnosed with breast or ovarian cancer. Four semi-structured focus-group interviews were conducted with 17 women recruited from a Norwegian multicenter study. The material was condensed, and conventional qualitative analysis was used to identify patterns in the participants’ descriptions. Three core themes were identified: 1) being “beside oneself” 2) altruism and ethical dilemmas 3) the need for support and counselling to assist the decision process. The present study indicates that women who are offered genetic testing when newly diagnosed with breast or ovarian cancer want a consultation with a health professional. Personalized support and counselling might empower women to improve their ability to manage and comprehend this overwhelming situation, and find meaning in this experience.

## Introduction

Most cases of breast and ovarian cancer are sporadic, while a minority are associated with inherited mutations in *BRCA1* or *BRCA2 (Alsop et al.* 2012 *; Kurian* 2010 *; Song et al.* 2014 *; Zhang et al.* 2011). For women carrying a mutation, the average lifetime risk of developing breast cancer may be up to 61 %, for ovarian cancer up to 59 % and for contralateral breast cancer up to 83 %. The risk varies across studies and populations (Brohet et al. 2013; Mavaddat et al. 2013; Møller et al. 2013). In addition to the considerable health risk, mutation carriers are confronted with difficult choices concerning risk management. They may choose regular surveillance, prophylactic mastectomy, prophylactic bilateral salpingo-oophorectomy and/or chemotherapy (van Oostrom and Tibben 2004). Studies show that carrying a mutation may have negative psychological implications (Beran et al. 2008; Di Prospero et al. 2001) including anxiety and depression (van Oostrom et al. 2003), anger and distress (Croyle et al. 1997), reduced quality of life, vulnerability and altered self-concept (Esplen et al. 2009), uncertainties about the future (DiMillo et al. 2013) and worry about cancer (Di Prospero et al. 2001). Testing for hereditary cancer may have extensive and irreversible consequences, and be more far-reaching than the patient can anticipate in advance (de Vries-Kragt 1998; Norwegian Ministry of Health and Care Services 2013). Women carrying a mutation have a 50 % risk of passing the mutation on to their children.

Traditionally, genetic testing has been based on referral of selected high-risk individuals to an extensive face-to-face genetic counselling session before and after the test (van Oostrom and Tibben 2004). However, as we approach a new era of personalized medicine and treatment-focused genetic-testing, this counselling procedure might be challenged (Komatsu and Yagasaki 2015; Trainer et al. 2010).

Concerns have been raised that rapid genetic testing at the time of diagnosis may create an undue psychological burden for women during a vulnerable time. Being diagnosed with severe illness might lead to despair and be a threat to a woman’s identity and to life itself (Hammer et al. 2009). The situation is emotionally and cognitively stressful and overwhelming (Gleeson et al. 2013; Vadamparampil et al. 2008; Ziliacus et al. 2012). However, research (Wevers et al. 2016) has found that rapid genetic counselling and testing in newly-diagnosed breast cancer patients did not have any measurable adverse psychosocial effects. Other studies have shown (Gleeson et al. 2013; Meiser et al. 2012; Ziliacus et al. 2012) that women recently diagnosed with breast and ovarian cancer found genetic testing as highly acceptable, primarily motivated by the fact that it can inform treatment options and possibly prevent family members from getting cancer. Some did not believe that receiving information about genetic testing would make the situation any worse (Gleeson et al. 2013; Ziliacus et al. 2012). Others wanted only brief information about the potentially increased risk of cancer, or no information at all. They felt there was enough to worry about, and that it might not be relevant to them anyway (Gleeson et al. 2013). Another study (Ardern-Jones et al. 2005) found that women believed that genetic testing would involve too much stress when they were already psychologically burdened.

A person’s capacity to respond to stress and stressful situations has been described by Aron Antonovsky (Antonovsky 1987). His salutogenic theory describes how coping, as a sense of coherence, may be created. Health is viewed as a continuum of more or less health, and the sense of coherence reflects the person’s capacity to respond to stressful situations and to improve the movement towards more health. Sense of coherence consists of three components: the perception of comprehensibility, manageability and meaningfulness. Comprehensibility refers to the extent to which a person perceives a certain stimulus as structured, predictable and explicable. Manageability expresses the extent to which the necessary resources to meet the demand are available, and meaningfulness is described as a challenge that is worth getting involved in. The components are intervened, and successful coping depends on the sense of coherence as a whole.

Reports about the impact of genetic testing in newly-diagnosed cancer patients are limited to studies of high-risk patients or other targeted groups (Meiser et al. 2011; Watts et al. 2012; Ziliacus et al. 2012), while studies on patients unselected for family history of *BRCA*-associated cancer and age of cancer diagnosis are lacking. More knowledge is needed to facilitate planning for optimal delivery of information and care to these women. The aim of this qualitative study was to investigate the experiences of women who had been offered and accepted genetic testing when newly diagnosed with breast or ovarian cancer.

## Methods

### Participants and Procedure

Participants were selected from a larger multi-center prospective study, DNA BONus (Høberg-Vetti et al. 2016), in which all women with newly diagnosed breast or ovarian cancer were consecutively offered testing for *BRCA1* and *BRCA2* mutations at the time of diagnosis, regardless of specific histologic features of their cancer, prior history of a related cancer, and/or family history of cancer. The women did not receive genetic counselling prior to genetic testing.

Respondents were identified from the list of participants in the prospective study. To secure a varied sample, women were recruited from two hospitals and strategically selected according to the following criteria: women of various ages and with breast cancer or ovarian cancer, a minimum of five months since they were diagnosed with cancer, completion of genetic testing and women with both negative and positive test results. The forty-two potential candidates received a written invitation. Twenty women gave their informed consent while three canceled before the interviews. Seventeen women attended the focus groups. Only one of the participants had tested positive for a *BRCA* mutation.

Ethical approval was obtained from the Regional Committees for Medical and Health Research Ethics (2012/62/REK vest). The women were informed that participation was voluntary and that they could withdraw at any time without any explanation. Names were altered to ensure anonymity.

### Data Collection and Analysis

In order to capture the women’s experiences, four focus group interviews were conducted during the last half of January 2014. A semi-structured interview guide was developed to explore women’s attitudes and preferences such as: timing of genetic information and testing, mode of delivery, information format, the situation when receiving information, and experiences about deciding whether to be tested. The women were encouraged to talk about their experiences and discuss issues beyond those highlighted by the moderator. By means of a questionnaire, demographic data was collected in advance (Table 1). The interviews lasted for about two hours and were conducted by the first author as moderator, and the last author as co-moderator. Both are nurses. The first author is a genetic counsellor, and the last author is a counsellor/PhD, experienced in qualitative research and cancer care.

**Table 1:** Characteristics of the study sample

Characteristics	
Total number of participants	17 women
Age in years	
40–49	5 women
50–59	7 women
60–69	5 women
Having biological children	16 women
Living	
- Alone	3 women
- With spouse/children	14 women
Diagnosed with breast cancer	13 women
Diagnosed with ovarian cancer	4 women
BRCA-mutation carrier	1 women
Time from diagnosis to interview	7–18 months (median 12)
Educational level	
- Less than high school	2 women
- High school or equivalent	8 women
- College/University	7 women
Currently employed	
- Full time	5 women
- Part time/ sick leave/ incapacitated for work	8 women
- Retired	4 women

The interviews were recorded and transcribed by the moderator, and analyzed in collaboration with the co-moderator according to Malterud (Malterud 2011) and systematic text condensation. The methodical approach consisted of four steps. First, the transcribed interviews were carefully read to gain a general understanding. Then, during multiple subsequent readings, important words and topics were identified and recorded as meaning units. Third, the colloquial language in each meaning unit was transformed into professional terminology, and the units were organized into main groups and sub-groups. Finally, all the meaning units were considered and synthesized into a condensed description, structured in themes describing the women’s experiences.

## Results

Three key themes were identified. All were related and illustrated aspects of the women’s experiences with genetic information and testing shortly after being diagnosed with cancer. Being diagnosed with cancer was itself a great burden. The women felt that their lives were turned upside down, and this made it difficult to receive information or comprehend everything that was happening. The findings revealed ambivalence and various reflections upon ethical dilemmas, related altruistically to the test. Most participants expressed that a consultation with a professional should be included when genetic testing is offered.

### Being “beside oneself”

After receiving their diagnosis, the women experienced their situation as shocking, chaotic and intense. Some described it as being “beside themselves”, as being disconnected and estranged from their own body. In addition to the practical things they had to deal with, they received a variety of information about e.g. cancer treatment, travel expenses, insurance, prostheses, wigs and research projects. Many of the participants felt overwhelmed by the amount of information.

Overall, the women experienced the diagnosis as an emotionally life-changing event. Furthermore, during the one or two weeks between diagnosis and surgery, even more information needed to be received and understood, decisions needed to be made and practical issues needed to be planned. Some tried to sort out the “important information” from the “less important”, while others left this to close family members. Julia said:

*In a way, I felt I was walking around with a sock over my head…and I just drifted along…because there was so much going on…*

Women reported difficulties coping with the information or handling everything that happened in a satisfactory way. One woman pointed to her head, stating that “…nobody was home.” Others described a feeling of “being completely beside themselves.” These are the circumstances under which the women received information about genetic testing that could determine whether the cancer was hereditary or not. Elisabeth said:

*I had my operation five days after I was diagnosed, and during those five days I was asked about the genetic testing. I was overwhelmed. I had to clean my desk at work, take a deep breath and mentally prepare for what was going to happen… It was too much for me to handle...and in addition, I had to decide whether to have a gene test or not.*

As the situation was already loaded with things to consider, some women did not find it problematic to add another consideration or another test at this stage. Gina was one of them:

*You feel you’re way up there…in a kind of bubble, and you’re in that bubble until you’re done with all the treatment…and then you descend…so whether you take a gene test or not doesn’t matter. There’s so much happening anyway. The more the better.*

Retrospectively some of the women acknowledged it was hard to remember details from this early, chaotic period. Nina interpreted this to be an outcome of the situation, as she was forced to focus on the most important issues. She said:

*It’s quite funny that there’s so much we can’t remember. I think you select…I mean, you focus on what’s important and what decisions you have to make, and the other things go out of sight.*

One woman could not remember whether she had given written consent to the test, because her mind was preoccupied at that time.

### Altruism and Ethical Dilemmas

A strong altruistic attitude appeared from the material. However, the question about performing genetic testing was not straightforward for all the women. It represented ethical dilemmas and challenges.

Genetic testing was seen as part of finding the exact diagnosis and helping close relatives to undergo adequate surveillance. The main motivation for the women to consent to testing was concern for their children. Monica said:

*The main reason for having a gene test was…my daughter…and I felt I had to do it…for her sake. Still, I was terrified that the test would be positive…and that I would have to tell her about it.*

The prospect of possibly preventing their children from having cancer was very important to the women, and without this element, the decision about the test would have been less clear. Gina stated:

*In this particular case it is possible to prevent, and that is very positive.*

Furthermore, she explained*:*

*The decision was easy for me. I just said yes. But I hadn’t really thought about what to do if the test had proved something was wrong…How would I have handled that?...I guess I just postponed those considerations. But it would really have been a new phase if the test had shown a bad result.*

The women’s reflections upon existential consequences of the testing seemed to vary. Apparently, several women agreed to the test without much hesitation. Retrospectively some reflected on how they would cope if a mutation was detected.

Some felt pressure from close family members to accept the test. Others spoke of their common responsibility to try to prevent more women dying of cancer by bringing them under surveillance, or by contributing to improved research. For some, however, the decision was challenging even though most of them basically felt positive about genetic testing. Johanna described it as a dilemma without any correct answers. She said:

*It’s an ethical dilemma. Nothing is really right or wrong…You just go for whatever you choose…and you’re doing it for those around you.*

Although they decided to reveal the test results to their children, they were also afraid to give information that could reduce their loved ones’ quality of life. The women tried to put themselves in their daughters’ place to make the best decision, and different aspects were weighed against each other. Several of the women with ovarian cancer communicated a deep concern about the possible outcome of the test. Johanna expressed her main concern as follows:

*My daughters would have to live with the consequences if the test result was bad …They are teenagers. It wasn’t an easy decision.*. .*Should I take the risk of putting them in that situation?*

Johanna was ambivalent about the test, but felt pressure from her oldest daughter who wanted the opportunity to prevent cancer. Unlike her daughter, she feared that information about an increased cancer risk could have a harmful effect. Several women found it hard to imagine what it would be like having knowledge of an inherited mutation. One assumed it could make recovery more difficult, because of the “threat of death the mutation implied.” Others feared seeing their children getting cancer and expressed relief when the test showed “good news.” Monica said:

*I was terrified that I might test positive and had to tell my daughter… and was really happy when I could tell her it was not hereditary.*

Some also feared that their children might neglect the recommended surveillance programs after being identified with a hereditary mutation. This, one woman said, would have been a “horrifying, unbearable situation”.

### The Need for Support and Counselling to Assist the Decision Process

The findings revealed the need for some sort of consultation with a professional. This was found to be essential to the women’s decision process, for different reasons.

All the women received an information letter from the main prospective study, while some in addition received oral information from a nurse or physician at the department responsible for their treatment. Most women expressed the wish for a consultation with a professional to assist their decision process, or to make the situation more manageable. The information letter was clear, but not sufficient due to the situation. The women thought a dialogue would be helpful to clarify any ambiguities. Therese said:

*I think a dialogue would have made the situation easier, at least for some of us… because we needed some complementary explanation to the information letter… not just the envelope to be opened when you get home. You have no idea of what you’ve received.*

The women asked for someone to “spend a few minutes” with them, to explain more deeply the implications of the test. They stressed the importance of being treated as individuals. Maria specified:

*See each woman individually…each woman and her fate is unique and could not be treated equally...*

Different reasons for wanting a face-to-face consultation were given, and those who had already had a dialogue with a doctor or nurse found it unthinkable not having this conversation. Susan said:

*When you’re in a state of shock, with a new diagnosis…and all you have to absorb…you can’t handle everything that’s coming to you. You must focus on just a little at a time, otherwise you’ll drown. I don’t think I would have been conscious of what this was if the doctors hadn’t talked to me about it in advance.*

The complexities of the genetic issue created a need for someone to talk to. Lina received oral information from the gynecologist, but realized that her understanding about the test and its consequences was still far from sufficient. She stressed the necessity of being “mentally clear” when receiving the information. Lina said:

*You go through phases…First the diagnosis and the shock, then the surgery…and then you recover from that. Then the information about how the surgery went. All the way there are stages where you are more or less amenable to information.*

A consultation would permit the women to ask questions spontaneously, to illuminate and clarify different aspects, and receive more and accurate information connected to the test. This was seen as crucial to continuing the decision-making process. Some women weighed the pros and cons several times as they found it difficult to make a final decision. One postponed the decision for a few months as she needed “… *time to think”*. Only one woman (Elisabeth) took advantage of the telephone counselling offered in the information letter. She made the call to

*…get confirmation of my own thoughts…to have some progress in my own decision process. I had no specific questions…Rather it was more that I found it hard to make a decision…I needed some support, and to share my thoughts with someone who knew what it’s all about…in order to make a decision... There was so much to consider within such a short time.*

According to Elisabeth, a supportive person would be:

*…someone who I can ask…and who can help me sort out my thoughts and put the problems to rest.*

Another woman was worried that the genetic testing could reveal unwanted information. She did not want to phone the counsellor as she considered her worries as unimportant. Telephone conversations were, by many, associated with something exhausting and energy-draining, and thus to be avoided in this period. Julia said:

*To me, the opportunity to call a genetic counsellor was kind of waste… I would never have phoned just to talk…or to get some more information…Never!*

While some women needed help to organize and sort out their own thoughts and reflections, others needed someone to sort out the information about the genetic testing from all the other information. Eva said:

*I think the oral information is essential. Because, if you only receive a letter, it might be easy just to put it away… that it becomes too difficult. Both the written and the oral information is incredibly important…because your ability to take in new information in this situation is limited…*

The participants considered the option to have a genetic test as significant. Thus, many assumed that better knowledge about its implications might lead to an increased number of women taking the test. According to the women, an obvious explanation for why many did not respond to the test was that the situation was already overloaded and stressful.

## Discussion

The “information overload” was quite a challenge for the women. Being diagnosed with a severe disease was shocking and the women experienced their situation as chaotic and intense. Some described “being beside themselves.” Although the prospect of preventing their children from having cancer was important, several women experienced the decision process concerning genetic testing as difficult. Others accepted the offer with hardly any considerations. All the participants reported that in retrospect they would have preferred a conversation with a health professional prior to genetic testing.

Many of the women described themselves as emotionally and mentally overloaded after their diagnosis. Being stricken by a severe disease is tough (Gleeson et al. 2013; Hammer et al. 2009; Vadamparampil et al. 2008; Ziliacus et al. 2012) and the women in our study found it difficult to integrate the information they received. Their capacity to make a decision about genetic testing during an already burdened situation raises important issues, and the circumstances should provide a framework for understanding the women’s experiences.

The women’s acceptance of genetic testing was primarily motivated by the prospect of protecting their children, and particularly their daughters. This altruistic attitude is known from Hallowell’s study (Hallowell et al. 2003), which found that most women previously diagnosed with breast or ovarian cancer generated genetic information for the benefit of others. Furthermore, her study revealed that women felt they really had no choice, because they had a moral obligation to clarify their genetic status and inform their family. The latter was also confirmed in our study. The women’s desire to prevent others from having cancer may, however, relate to the description of meaningfulness, described by Antonovsky (Antonovsky 1987). To find meaning in the situation is seen as a motivating component, essential for successful coping. Although the word ‘meaningfulness’ was not used directly, the interviews implied that helping others was experienced as meaningful to the women. Our study was conducted before genetic testing was used to inform treatment options in Norway, which could explain why the women did not discuss treatment option as a reason for genetic testing.

Deciding about genetic testing generated challenging and conflicting thoughts and emotions in the women, particularly regarding their daughters’ future health. Some women made their choice without much hesitation or consideration of the consequences. Others described the decision as an ethical dilemma between alternatives that seemed neither right nor wrong. The women definitely wanted to prevent their children from getting cancer but feared that the test results might reduce their children’s quality of life. Knowing about an inherited mutation may have negative psychological consequences and the uncertainties associated with the mutation, such as the constant fear of getting cancer, the fear for one’s children and uncertainties regarding womanhood, may be hard to tackle because of the potentially severe outcome (DiMillo et al. 2013). By considering the test, the women in our study were introduced to issues that could lead to extensive and irreversible consequences for themselves and their relatives. The ambivalence between disclosing genetic information and caring for one’s family has been described elsewhere (Hallowell et al. 2003).

Our data identified the women’s need to consult health professionals with adequate knowledge about hereditary cancer. This echoes previous findings that newly-diagnosed patients wanted information about treatment-focused genetic testing via face-to face consultation. Written information was seen as supporting material (Meiser et al. 2012), and considered inadequate as the only information about genetic testing (Vadamparampil et al. 2008). As our findings revealed, the women wanted a consultation with a professional who could help them sort out their own thoughts and feelings about genetic testing. For future interventions, the women were convinced that this would be invaluable to help handle and understand the genetic information. Lode and colleagues (Lode et al. 2012) describe similar findings in studies concerning shared decision-making in women undergoing risk-reduction procedures due to genetic constitution. Expressing their feelings and thoughts in a conversation with a health professional increased the women’s understanding and was emphasized as more important than factual knowledge about the surgery. Moreover, Hammer and colleagues (Hammer et al. 2009) found that health personnel play an invaluable role, as just being present with the patients might trigger hope and activate internal strength. In our study, women who participated in a consultation felt they were taken care of and were more conscious of the implications of the test.

Providing some consultation could be seen as a way of supporting the women’s comprehensibility and manageability, as defined by Antonovsky (Antonovsky 1987). A tailored consultation with a health professional could meet the women’s needs and actually help them find meaning in their situation. Coping strategies may be created, and further determine the women’s abilities to regain health or strength. By strengthening women’s sense of coherence, Antonovsky’s theory (Antonovsky 1987) claims that they will cope better with challenges.

Our study focuses on a new group of patients who must understand and decide on complicated issues in a context of chaos and numbness. These women might be less prepared that cancer can be hereditary, and the fact that they are not adequately informed prior to the test could make them more vulnerable to stress, compared to those who traditionally seek genetic testing (Croyle et al. 1997). To accept the test without insight about its consequences is ethically challenging to women’s coping abilities, and to informed consent. Moreover, if a woman declines genetic testing because she is not sufficiently informed and therefore miss out an opportunity to inform her family, this may represent another ethical challenge.

We are in need of educational resources or guidelines, which specifically help newly-diagnosed patients with the decision-making process (Meiser et al. 2012; Watts et al. 2012). The personalized approach seems to be particularly important to meet different requests for support and counselling. This tailored counselling might be a better tool for empowering patients, rather than more standardized information. If the need for increased knowledge and better coping strategies is not met, rapid genetic testing could be an ethical challenge, and inflict an increased burden on some women.

### Strengths and Limitations

A major strength of the study is its contribution to a small body of literature on rapid genetic testing for hereditary breast or ovarian cancer. The sample had a favorable composition and included women from different hospitals, with breast cancer and ovarian cancer, thus diversifying the perspectives of the findings. Participants were selected from an ongoing multi-center study and had accepted genetic testing at time of diagnosis. This might favor certain characteristics among the participants. However, the findings may underscore women’s experiences and needs when faced with genetic testing during a state of chaos. Another limitation is that only one of the women was diagnosed with a *BRCA* mutation.

### Research Recommendations

Based on the limitations of this study, further research, including more mutation carriers and women who abstain from testing, might provide more insight into the debate about genetic testing of newly-diagnosed cancer patients. Such an understanding might be essential in order to provide instructions for personalized genetic counselling to this group of patients.

### Practice Implications

A consultation with a health professional might be crucial to the coping process, and should be routinely implemented when genetic testing is offered to this group or similar groups of patients in the future. A consultation may increase a patient’s comprehensibility by giving qualified and personalized counselling about the testing and its implications. It is important, however, to respect the patient’s own resources for handling the situation so the information does not become an extra burden. Women’s manageability may be promoted and maintained by navigating them through the information. This may also decrease the level of tension and stress these women experienced, and finally support them while deciding about genetic testing.

A consultation with a health professional requires that more healthcare disciplines prepare for the integration of genetics into routine care. The consultation provides opportunities for detecting vulnerable patients in order to personalize their care and support.

## Conclusion

On the basis of the women’s stories we have identified different needs which must be met to provide an optimal treatment, care and support of women who are offered testing for hereditary cancer shortly after cancer diagnosis. The women in the study expressed a need for clarification connected to genetic testing, help in sorting out the important from the less important information, thus making the situation more manageable, and to be seen as individuals receiving individualized information. Our findings suggest that a face-to-face consultation with a health professional qualified in medical genetics seem to be essential to increase women’s knowledge and empower them in their decision-making process.
